# Pathomimie de l'enfant: à propos d'une observation

**DOI:** 10.11604/pamj.2013.14.23.1293

**Published:** 2013-01-16

**Authors:** Rachid Abilkassem, Nezha Dini, Hakim Ourai, Mohamed Kmari, Aomar Agadr

**Affiliations:** 1 Service de pédiatrie, Hôpital Militaire d'Instruction Mohamed V, Rabat, Maroc

**Keywords:** Dermatose auto provoquée, dermatose factice, pathomimie, self-induced dermatitis, Dermatitis Artefacta, pathomimie

## Abstract

La pathomimie cutanée se définit comme une maladie factice, provoquée dans un etat de conscience claire par le patient lui-même, au niveau du revêtement cutanéo-muqueux et/ou des phanères. Rare chez l'enfant, il s'agit d'une manifestation psychopathologique potentiellement grave et souvent difficile à prendre en charge. Nous rapportons le cas d'une fillette de 10 ans présentant une pathomimie sous forme de lésions excoriées multiples du visage.

## Introduction

Les pathomimies sont définies comme des troubles factices entièrement provoqués dans un état de conscience claire par le sujet lui-même sur son propre corps. On ne retrouve pas de motif rationnel précis pouvant expliquer une telle conduite pathologique, ce qui distingue cette dernière de la simulation. Enfin, le patient dissimule sa responsabilité dans la survenue de ses troubles aux différents soignants interpellés. C'est une manifestation qui est fréquemment méconnue d'où le retard diagnostic et l'escalade des examens paracliniques inutiles.

## Patient et observation

Il s'agit d'une fillette de 10 ans, deuxième d'une fratrie de trois, scolarisée. L'histoire de la maladie remontait à l'âge de 4 ans par l'apparition d'excoriation au niveau du visage et des membres. La mère rapportait aussi la notion d'énurésie primaire. L'enfant est était d'humeur triste, sans entrain.

**Figure 1:**
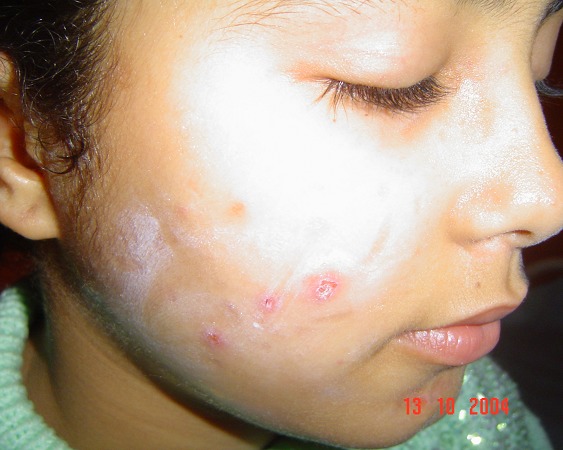
Excoriations des joues, à limites nettes, sur peau saine.

**Figure 2:**
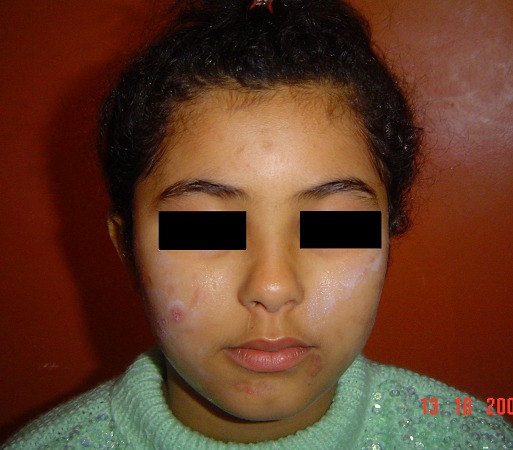
Cicatrices pigmentées du menton.

L'examen physique objectivait des excoriations multiples au niveau des deux joues, bien limitées, à bords nets, sur une peau saune, certaines en coup d'ongle, avec des cicatrices pigmentées d'anciennes lésions de prurigo (Figure 1, Figure 2).

L'interrogatoire répété de la petite fille et de la mère retrouvait la notion de conflits familiaux (notamment avec ses frères), ainsi que la promiscuité au niveau du domicile familial (oncles et leurs enfants vivant sous le même toit). L'examen par un pédopsychiatre, confronté aux données cliniques a conclut à des lésions de pathomimie au niveau du visage, et des lésions de prurigo manipulées au niveau des membres. Elle a reçus des soins locaux ainsi que du Bromo-galactogluconate de calcium (Calcibronat^®^). La patiente a malheureusement a été perdue de vue.

## Discussion

De Grec pathos: “souffrance”, et mimos: “qui imite”, le terme de pathomimie a été créé en 1908 par Paul Bourget et Dieulafoy pour désigner un état morbide voisin de la mythomanie, caractérisé par le besoin qu'éprouvent ceux qui en sont atteints de simuler une maladie, parfois même au prix d'une automutilation [1]. Le diagnostic de pathomimie est toujours difficile à établir parce que tous les organes peuvent être concernés par la conduite pathologique. Les troubles factices ne sont pas exceptionnels chez l'enfant et leur fréquence est certainement sous-estimée, leur diagnostic étant très souvent retardé [2]. Les troubles factices surviennent chez l'enfant en deux circonstances:

1. Le trouble factice est créé par l'enfant lui-même sur son propre corps: c'est la classique pathomimie.
2. Le trouble factice est créé sur le corps de l'enfant par un sujet extérieur, la mère le plus souvent: c'est, selon la dénomination de Meadow, le syndrome de Münchhausen dit par procuration.

Les pathomimies cutanées sont les plus fréquentes, peut être parce que la peau est un organe visible et facile à atteindre. Elles surviennent surtout chez les petites filles prépubères et leurs aspects cliniques sont très variés selon les moyens utilisés par ces enfants [2].

Les lésions siègent le plus souvent au niveau des parties découvertes, offertes à la vue (visage, cou, mains, avant-bras). Les aspects cliniques peuvent être de types divers; excoriations, stries de grattage, ulcération créées par des agents physiques ou chimiques; lésions infectieuses cutanées à type d'abcès ou de cellulites par injection de matériel souillé, granulomes unguéaux, lymphoedème par restriction chronique d'un membre, contusion, lésion eczématiformes [1, 3, 4]. Dans notre cas, les lésions siègeaient sur les joues et certaines étaient en coups d'ongle. Classiquement, il s'agit de lésions d'apparition brutale, d'emblée sur peau saine, aux bord bien limités et aux contours géographiques, dont la cicatrisation est trainante, et d'évolution capricieuse, émaillée de surinfections [1, 3–5]. Parfois la mise hors d'accès des lésions par bandage ou pansement peut amener leur guérison rapide [6]. Il faut noter en plus une forme clinique particulière et de meilleur pronostic: la dermatose auto-aggravé (par exemple des récidives d'eczéma par réexposition au produit allergisant). Notre patiente présentait des lésions de prurigo qui sont habituellement prurigineuse, et qu'elle manipulait certainement.

Il faut distinguer des pathomimies cutanées toutes les autres manipulations qui prennent le revêtement cutanéo-muqueux et/ou les phanères pour cible mais qui ne sont pas tenues secrètes par le sujet: la trichotillomanie (consiste en l'arrachage par l'enfant de ses propres cheveux et/ou poils), les excoriations dites névrotiques, les automutilations qui se rencontrent chez les enfants souffrant de grave désordres de la personnalité. Le diagnostic est évident et la plupart des troubles factices peuvent entrainer, surtout si le délai diagnostique est long, des complications somatiques graves, parfois même mortelles: accidents infectieux à répétition, amputation, actes chirurgicaux itératif et délabrant'.

Les pathomimies de l'enfant surviennent le plus souvent dans un milieu familial en crise (séparation, divorce') ou/et dans une situation d'échec scolaire. Ainsi, elles réalisent fréquemment un véritable appel à l'aide. Dans ces cas le pronostic est bon et l'amélioration survient dès que le problème conflictuel est mise à jour et qu'une réponse affective est donnée à l'enfant. Notre malade patiente qui présentait par ailleurs une énurésie, vivait dans un milieu familial conflictuel, et elle exprimait son mal-être par des excoriations visibles sur son visage. Dans de rare cas, l'enfant persiste dans sa conduite pathologique, il faut alors craindre l'existence d'un état dépressif ou même de grave s trouble de personnalité qui demanderont une longue prise en charge psychiatrique avant toute amélioration somatique.

Même si la pathomimie de l'enfant est de meilleur pronostic que celle de l'adulte, elle réalise toujours un problème thérapeutique complexe.

Le principal objectif thérapeutique est psychologique, il faut donc savoir arrêter les investigations lourdes ainsi que l'escalade thérapeutique. Il est important de ne pas chercher la confrontation directe avec l'enfant, et de ne pas rechercher le flagrant délit ou l'aveu à tout prix (risque de surenchère somatique, de décompensation psychologique). Le médecin doit faire en sorte d'aborder avec l'enfant

Le contexte psychologique dans lequel la maladie est apparue, faisant ainsi comprendre à l'enfant qu'il peut envisager qu'une souffrance psychique puisse s'exprimer au niveau du corps et que son rôle n'est pas de juger la façon dont cette souffrance psychique s'exprime mais d'aider l'enfant à se débarrasser de cette dernière [2].

Par ailleurs, un entretien explicatif et déculpabilisant avec las parents est indispensable. Le recours au pédopsychiatre intervient en second lieu, après avoir tissé un lien de confiance avec l'enfant, afin d'éviter une fuite ou un nomadisme médical.

La psychothérapie réalisée est, le plus souvent, une psychothérapie dite de soutien, et les antidépresseurs sont indiqués si un état dépressif est diagnostiqué.

Pour le traitement dermatologique, peuvent être proposés: le pansement occlusif à visée diagnostique (mise hors d'accès des lésions) et thérapeutique, et selon les cas: Antiseptiques, antibiotiques locaux ou généraux et le émollients [6].

## Conclusion

Les pathomimies cutanées de l'enfant sont rares, de diagnostic souvent tardif et d'évolution longue et capricieuse. Si leur pronostic est en général meilleur que chez l'adulte, leur prise en charge est difficile est décevante. Enfin, pour éviter des sentiments de frustration et d'agressivité envers le patient, le médecin ne doit jamais oublier que la pathomimie est le résultat d'un conflit intrapsychique qui dépasse le patient lui-même.
